# Recent Advances in Ionic Mechanisms in Pituitary Cells: Implications for Electrophysiological and Electropharmacological Research

**DOI:** 10.3390/jcm14093117

**Published:** 2025-04-30

**Authors:** Sheng-Nan Wu, Ya-Jean Wang, Zi-Han Gao, Rasa Liutkevičienė, Vita Rovite

**Affiliations:** 1 Department of Research and Education, An-Nan Hospital, China Medical University, No. 66, Section 2, Changhe Road, An Nan District, Tainan 70965, Taiwan; 2 Institute of Basic Medical Sciences, National Cheng Kung University Medical College, Tainan 701401, Taiwan; 3 Department of Senior Services Industry Management, Minghsin University of Science and Technology, Hsinchu 300401, Taiwan; 4 Neuroscience Institute, Medical Academy, Lithuanian University of Health Sciences, Eiveniu 2, 50106 Kaunas, Lithuania; 5 Latvian Biomedical Research and Study Centre (BMC), LV-1067 Riga, Latvia

**Keywords:** pituitary cell, voltage-gated ionic currents, small-molecule modulators

## Abstract

Pituitary cells are specialized cells located within the pituitary gland, a small, pea-sized gland situated at the base of the brain. Through the use of cellular electrophysiological techniques, the electrical properties of these cells have been revealed. This review paper aims to introduce the ion currents that are known to be functionally expressed in pituitary cells. These currents include a voltage-gated Na^+^ current (*I*_Na_), erg-mediated K^+^ current (*I*_K(erg)_), M-type K^+^ current (*I*_K(M)_), hyperpolarization-activated cation current (*I*_h_), and large-conductance Ca^2+^-activated K^+^ (BK_Ca_) channel. The biophysical characteristics of the respective ion current were described. Additionally, we also provide explanations for the effect of various drugs or compounds on each of these currents. GH_3_-cell exposure to GV-58 can increase the magnitude of *I*_Na_ with a concurrent rise in the inactivation time constant of the current. The presence of esaxerenone, an antagonist of the aldosterone receptor, directly suppresses the magnitude of peak and late *I*_Na_. Risperidone, an atypical antipsychotic agent, is effective at suppressing the *I*_K(erg)_ amplitude directly, and di(2-ethylhexyl)-phthalate suppressed *I*_K(erg)_. Solifenacin and kynurenic acid can interact with the K_M_ channel to stimulate *I*_K(M)_, while carisbamate and cannabidiol inhibit the *I*_h_ amplitude activated by sustained hyperpolarization. Moreover, the presence of either rufinamide or QO-40 can enhance the activity of single BK_Ca_ channels. To summarize, alterations in ion currents within native pituitary cells or pituitary tumor cells can influence their functional activity, particularly in processes like stimulus–secretion coupling. The effects of small-molecule modulators, as demonstrated here, bear significance in clinical, therapeutic, and toxicological contexts.

## 1. Introduction

The pituitary gland, commonly known as the “master gland”, plays a crucial role in regulating a wide range of physiological processes by secreting several important hormones. It consists of two main sections: the anterior and posterior pituitary. The anterior pituitary contains various cell types, including somatotrophs, thyrotrophs, corticotrophs, gonadotrophs, melanotrophs, and lactotrophs. Among these, lactotrophs are a specific cell population responsible for secreting prolactin.

By utilizing specialized separation techniques [1], it is possible to immortalize pituitary cells derived from pituitary tumors, enabling in-depth academic research into the functional properties of these cells. Additionally, unlike conventional intracellular recordings that involve impaling or piercing the cell, the patch-clamp technique, which uses a relatively large electrode tip with a pipette resistance of 2–4 MΩ, can be used on small cells with minimal damage, making it suitable for cellular electrophysiological experiments [2,3]. In other words, the electrode used in patch-clamp recordings applies negative pressure to generate suction, which holds the small cell, such as a pituitary cell, in place or keeps it in a relatively suspended position without displacing or distorting it. This negative pressure is applied to securely and firmly attach the cell to the measuring electrode and generate a sealing resistance greater than 1 GΩ (1 × 10^9^ ohms) [2].

**Figure 1:**
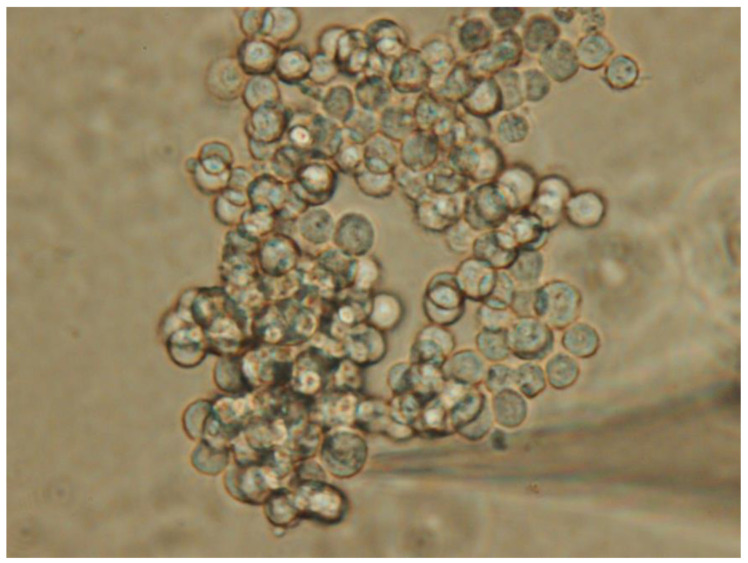
Morphology of GH_3_ pituitary tumor cells. Live cell imaging was captured using an inverted microscope at a magnification of ×200. The horizontal, elongated, triangle-shaped shadow in the bottom right corner indicates that the measuring electrode is approaching the cell. To perform patch-clamp recordings, the patch electrode was gradually positioned against the pituitary cells using a micromanipulator. Despite being dispersed in the recording chamber, the pituitary cells, which are slightly uniform in size, show a tendency to aggregate.

With this method, when the electrode gradually approaches and comes into contact with the cells, the cells sometimes move away from the electrode. Unlike conventional fine microelectrodes, the tip diameter of patch electrode is wider, so when approaching the cell, some electrolyte-containing liquid may leak out, which could slightly stimulate the cell. Additionally, if the electrode operation fails, the electrode’s surface becomes contaminated due to contact with the cell, and the used electrode cannot be reused. Moreover, a stable anti-vibration table and the proper use of a fine-tuning micromanipulator are crucial for accurate potential or current recordings [2]. Nonetheless, this procedure eliminates the need to impale or puncture the cell, reducing cell damage and enhancing electrophysiological recordings. Figure 1 illustrates a typical population of pituitary tumor cells, specifically GH_3_ cells, with an electrode approaching them. In this paper, we review the main ion currents in pituitary cells that are influenced by changes in cell membrane voltage, exploring their physiological and pharmacological implications.

## 2. Physiological Importance and Pharmacological Impact on Voltage-Gated Ionic Currents in Pituitary Cells

**Figure 2:**
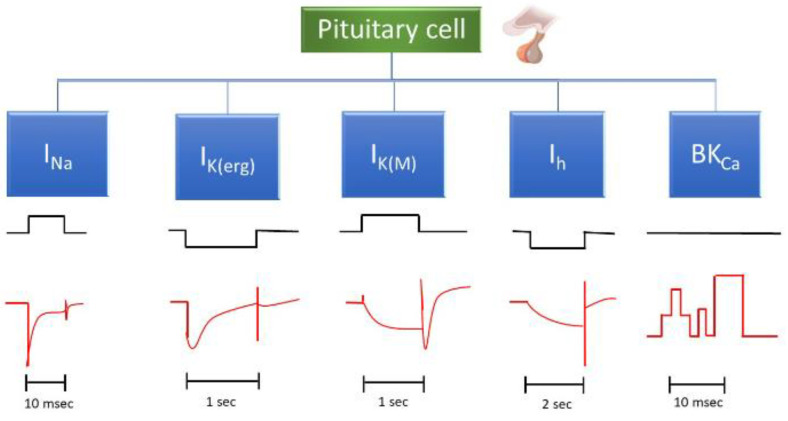
Main ionic currents observed in pituitary cells, along with their corresponding voltage and current traces. The full names of these ion currents can be found in the abbreviation section of the main text. The black and red traces represent voltage and the respective ion currents. The y-axis scales for each current are indicated below the respective traces. For measurements of *I*_K(erg)_ or *I*_K(M)_, the cells are bathed in a high K^+^ solution (145 mM K^+^). As shown in black color, an upward voltage step denotes depolarization, while a downward step represents hyperpolarization. The detailed voltage-step protocol applied to the membrane potential requires referencing of various ionic currents. Downward current (red color) indicates inward current, meaning Na^+^ or K^+^ ions are entering the cell. Of note: “BK_Ca_” refers to single channel activity, reflecting the opening or closing of BK_Ca_ channel in the cell membrane, while the other currents represent whole-cell ionic currents.

In this paper, we will provide a detailed description of the potential voltage-gated currents that may occur in pituitary cells and discuss various drugs or compounds that are currently known to influence these currents (Table 1). Figure 2 illustrates the primary voltage-gated ionic currents typically observed in pituitary cells. We also present examples of various drugs or compounds that influence the amplitude, gating properties, and voltage-dependent activation, inactivation, or deactivation of these ion currents.

*A.*  *Voltage-gated Na^+^ current (I_Na_)*

The Na_V_ channels exist in nine isoforms (Na_V_1.1-1.9, also known as *SCN1A-SCN5A* and *SCN8A-SCN11A*) and are found in various mammalian excitable tissues, including the central and peripheral nervous systems, as well as the neuroendocrine or endocrine systems [17,18,19,20]. When activated, Na_V_ channel activity generates a macroscopic *I*_Na_, which is characterized by its rapid activation and inactivation, both occurring within milliseconds. This current temporarily depolarizes the membrane, providing positive feedback that triggers the upstroke of the action potential (AP). As a result, changes in the magnitude of *I*_Na_ can influence the amplitude, frequency, and patterns of APs in various electrically active cells [17,20,21]. Previous studies have shown that Na_V_ channels are present in all secretory pituitary cells, including pituitary GH_3_ cells [20]. Additionally, late (or persistent) *I*_Na_ has been progressively identified, and its presence exerts a significant influence on the electrical behavior and firing patterns of pituitary cells [5,20,22,23,24].

**1.**  **GV-58 ((2R)-2-[(6-{[(5-methylthiophen-2-yl)methyl]amino}-9-propyl-9H-purin-2-yl)amino]butan-1-ol)**

GV-58 was developed as a modification of (R)-roscovitine. It has been viewed as an opener of N- and P/Q-type Ca^2+^ channels [25,26,27,28,29]. This compound was presumably thought to slow the closing of the voltage-gated Ca^2+^ (Ca_V_) channel, resulting in a large increase in total Ca^2+^ entry during motor nerve AP activity [30,31]. Its presence was reported to enhance spontaneous and evoked activity from the cultures of murine ventral horn of the spinal cord on microelectrode arrays [25]. Earlier studies have demonstrated that this compound is effective for the management of neuromuscular weakness, such as Lambert–Eaton myasthenic syndrome [26,27,32,33,34].

However, recent studies have demonstrated that, as pituitary GH_3_ cells were continually exposed to GV-58, the peak and late components of *I*_Na_ activated by abrupt step depolarization were increased in a concentration-, time-, and state-dependent manner [4]. The *I*_Na_ activated by brief depolarizing pulse was sensitive to either block by tetrodotoxin or stimulation by GV-58, but it failed to be affected by ω-conotoxin MVIID. ω-Conotoxin MVIID, a small, disulfide-rich peptide purified from the venoms of predatory cone snails, was reported to be an inhibitor of N- and P/Q-type Ca^2+^ currents in adrenal chromaffin cells [35]. The recovery of *I*_Na_ inactivation induced with varying interpulse intervals was overly enhanced in the presence of GV-58. The decline of peak *I*_Na_ during rapid repetitive stimuli was slowed during cell exposure to GV-58. This compound stimulated peak *I*_Na_ in a tonic and use-dependent manner. GH_3_-cell exposure to GV-58 was also found to enhance the magnitude of instantaneous resurgent and window *I*_Na_. Under current-clamp conditions, GV-58 effectively increased the frequency and spontaneous APs. The NSC-34 motor neuron-like cells could be enhanced by adding GV-58. The mRNA transcripts for the α-subunit of Na_V_1.1, Na_V_1.2, and Na_V_1.6 were reported to be expressed in pituitary GH_3_ cells [21]. Findings from this study can be interpreted to reflect that GV-58 can interact with Na_V_ channels to stimulate the magnitude and to alter the gating of *I*_Na_ [4]. These results would engage in the modification of spontaneous APs in electrically excitable cells like GH_3_ and NSC-34 cells, presuming that similar in vitro or in vivo findings occur.

Concentration-dependent stimulation of both peak and late *I*_Na_ was observed with GV-58, and it exhibited effective half-maximal concentration (EC_50_) values of 8.9 and 2.6 μM for peak and late *I*_Na_, respectively [4]. Continuous exposure to GV-58 resulted in an increase in the amplitude of peak *I*_Na_ in response to abrupt step depolarization, accompanied by a significant increase in the value of slow component of *I*_Na_ inactivation time constant. It is therefore important to emphasize caution when ascribing the expanding use of GV-58 to its selective agonistic effects on N- and P/Q-type Ca_V_ channels [27,32,33].

**2.**  **Esaxerenone (ESAX, Minnebro^®^, CS-3150, XL-550, (4S)-4-(5,6-dimethyl-2-oxo-1,2-dihydropyridin-3-yl)-2-fluoro-N-(1-methyl-1H-pyrazol-4-yl)benzamide)**

ESAX, known to be a newly oral, non-steroidal selective blocker on the activity of mineralocorticoid receptor, has been growingly used for the management of varying pathologic disorders, such as primary aldosteronism, refractory hypertension, chronic kidney disease, diabetic nephropathy, and heart failure [36,37,38,39,40]. Alternatively, the activity of mineralocorticoid receptor has been previously demonstrated in pituitary cells including GH_3_ cells or in various brain regions [41,42,43,44].

However, earlier studies have shown the ability of ESAX to suppress the peak and late component of *I*_Na_ elicited during short depolarizing pulse in pituitary GH_3_ cells [5]. The subsequent addition of ESAX reversed tefluthrin-mediated increase in the strength of voltage-dependent hysteresis of persistent *I*_Na_ activated by the isosceles-triangular ramp pulse. Tefluthrin was known to be an activator of *I*_Na_ [45]. In pituitary MMQ cells, the presence of ESAX was effective at decreasing the amplitude and gating of *I*_Na_.

**Figure 3:**
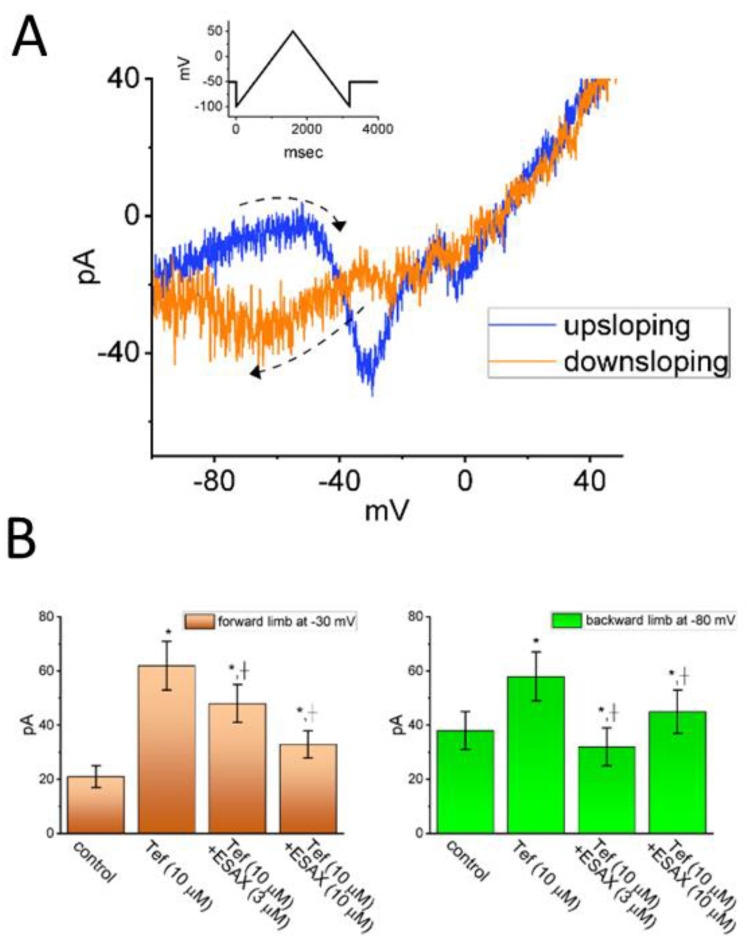
Effect of ESAX on voltage-dependent hysteresis of persistent *I*_Na_ (*I*_Na(P)_) in GH_3_ cells. (**A**) Figure-of-eight pattern in voltage-dependent hysteresis of *I*_Na(P)_ activated by isosceles-triangular ramp voltage with a total ramp duration of 3.2 s (or a ramp speed of ±0.094 mV/ms) in the presence of 10 μM tefluthrin (Tef), an activator of *I*_Na_. The ascending limb is shown in blue, while the descending one is shown in orange. An inset at the top illustrates the applied double ramp voltage. The dashed arrow indicates the direction of the current trajectory as time progresses. (**B**) Summary bar graph showing the effect of Tef (10 μM) and Tef (10 μM) plus ESAX (10 μM) on *I*_Na(P)_ amplitude activated by the upsloping and downsloping limb of triangular ramp pulse (mean ± SEM; n = 7 for each bar). The current amplitude on the left side was taken at the level of −40 mV, corresponding to the forward (upsloping) limb of triangular pulse, which was used to evoke *I*_Na(P)_ (i.e., high-threshold *I*_Na(P)_). On the right side, the current amplitude was measured at −80 mV, during the backward (downsloping) phase of the pulse, corresponding to low-threshold *I*_Na(P)_. * Significantly different from control (*p* < 0.05) and + significantly different from Tef (10 μM) alone group (*p* < 0.05). This figure is adapted from Chang and Wu [5] and published under the terms and conditions of the Creative Commons Attribution (CC BY) license.

The IC_50_ value needed for ESAX-mediated inhibition of peak or late *I*_Na_ observed in GH_3_ cells was yielded to be 13.2 or 3.2 μM, respectively, the value of which was distinguishable between its suppressive effects on these two components of the current [5]. The presence of neither dexamethasone nor aldosterone affected the magnitude or gating of *I*_Na_. With continued exposure to aldosterone, further addition of ESAX was still able to suppress peak *I*_Na_. It therefore seems unlikely that ESAX-mediated inhibition of *I*_Na_ amplitude together with changes in the gating kinetics of the current was predominantly associated with its blockade of mineralocorticoid receptor. Moreover, as shown in Figure 3, the presence of ESAX (10 μM) can effectively diminish the strength of voltage-dependent hysteresis of persistent *I*_Na_ (*I*_Na(P)_) activated by prolonged isosceles-triangular ramp voltage observed in GH_3_ cells [5].

*B.*  *erg-mediated K^+^ current (I_K(erg)_)*

The *erg* (ether-à-go-go related gene)-mediated K^+^ current (*I*_K(erg)_), which is encoded by three different subfamilies of the *KCNH* gene, is enabled to generate the pore-forming α-subunit of *erg*-mediated K^+^ (i.e., K_erg_ or K_V_11) channels [46,47]. These K^+^ currents are widely believed to represent the cloned equivalent of the rapidly activating delayed-rectifying K^+^ currents found in cardiac myocytes, and the *KCNH2* gene encodes the α-subunit responsible for pore formation in K_V_11.1 channels, commonly referred to as the human *erg* K^+^ (HERG) channels [47,48,49]. The intrinsic presence of *I*_K(erg)_ extends beyond excitable cells to various types of epithelial or neoplastic cells, as reported previously [48,49]. Earlier work has also shown the effectiveness of *I*_K(erg)_ magnitude in regulating the apoptosis and proliferation of various types of neoplastic or stem cells [47,49,50].

**1.**  **Risperidone (Risperdal^®^, 3-[2-[4-(6-fluoro-1,2-benzisoxazol-3-yl)piperidin-1-yl]ethyl]-2-methyl-6,7,8,9-tetrahydro-4H-pyrido[1,2-a]pyrimidin-4-one)**

Risperidone, a benzisoxazole compound approved for use in the United States in 1994, is recognized for its effectiveness in terminating acute psychotic episodes and preventing their recurrence in patients with schizophrenia [51,52]. However, the significant and consistent neuroendocrine effect of neuroleptic drugs including risperidone is to stimulate prolactin secretion and cause galactorrhea, although these untoward effects vary greatly in potency and chemical structure [52,53,54]. The important site of action has been thought to be due to the blockade of dopamine D2 and 5-HT receptors [51,55]. However, other evidence suggests that these neuroleptics, including risperidone, may cause a significant prolongation of electrocardiographic QTc interval [56,57].

In a previous study of pituitary GH_3_ cells, the exposure to risperidone effectively suppressed the magnitude of *I*_K(erg)_, together with slowing the rate of activation [7]. The results also showed a difference in the reciprocal time constants of *I*_K(erg)_ decay at various voltages, implying that risperidone may enhance the rate of deactivation [7]. It is therefore possible that the different levels of membrane potential can exert an interaction with *erg*-mediated K^+^ (K_erg_) channels to modify the magnitude and gating of whole-cell *I*_K(erg)_ in GH_3_ cells. In other words, the sensitivity to risperidone in pituitary lactotrophs would be dependent on the preexisting level of resting membrane potential, the firing rate of AP, or the concentration of risperidone used, assuming that the risperidone action in pituitary lactotrophs is similar to that in GH_3_ cells [7].

It is worth noting that neither dopamine nor metoclopramide affected the magnitude of *I*_K(erg)_ in GH_3_ cells, although haloperidol and thioridazine mimicked the risperidone-mediated inhibition of *I*_K(erg)_. Metoclopramide was reported to antagonize the dopamine receptor. In GH_3_ cells preincubated with dopamine, the inhibitory effect of risperidone on *I*_K(erg)_ remained unchanged. Several tyrosine kinase inhibitors were also found to suppress the *I*_K(erg)_ magnitude [58]. Therefore, the effect of risperidone on *I*_K(erg)_ appears to be direct and independent of its binding to dopamine receptors. Care may need to be taken in ascribing the risperidone-mediated prolactin release or QT prolongation to the blockade of dopamine receptors residing in vivo or in vitro [6,51,59]. These findings imply that the risperidone-mediated stimulation of prolactin release could be partly, if not entirely, ascribed to the direct blockade of *I*_K(erg)_ functionally expressed in pituitary lactotrophs [6,7,54].

**2.**  **Di(2-ethylhexyl)-phthalate (DEHP)**

Phthalates are a group of chemicals that are mainly used as plasticizers to allow stiff plastics, such as polyvinyl chloride, to become more flexible. Because phthalate plasticizers are not chemically bound to polyvinyl chloride, they may leach, migrate, or evaporate into air and atmosphere, foodstuffs, and other materials [60,61]. Due to their suitable properties and low cost, the general population will become significantly exposed to these compounds [62]. One of the phthalate plasticizers used in a wide variety of medical devices is di(2-ethylhexyl)-phthalate (DEHP), which is recognized to be an endocrine-disrupting chemical [62,63,64,65,66,67].

Previous work has shown that high doses of DEHP could change cell size or function in the anterior pituitary gland [64,67]. DEHP was demonstrated to suppress tamoxifen-induced apoptosis, possibly linked to its estrogenic effects, as well as to influence signaling pathways in pituitary GH_3_ cells [68,69]. This compound was also noted to have an age-dependent influence on the pituitary–adrenocortical axis in vivo [67]. A previous report revealed that the concentration of phthalate esters, including DEHP, in semen or serum samples was positively associated with circulating prolactin levels in adult men [70]. DEHP was found to impair the electrical and mechanical behavior of the cardiac cell network [71].

The study conducted by Wu et al. [8] revealed that DEHP has been observed to reduce the amplitude of *I*_K(erg)_ in pituitary GH_3_ cells in a concentration-dependent manner, with an IC_50_ value of 16.3 μM. This IC_50_ value is notably lower than the typical concentration of DEHP found in human blood or blood components, which has been reported to range from 10 to 650 μg/mL (equivalent to 27 μM to 1.6 mM) [72]. Exposure of GH_3_ cells to DHEP was found to alter the activation kinetics of *I*_K(erg)_ without affecting the deactivation kinetics of the current. Additionally, the presence of DEHP led to an increase in the firing of spontaneous APs in these pituitary cells.

**Figure 4:**
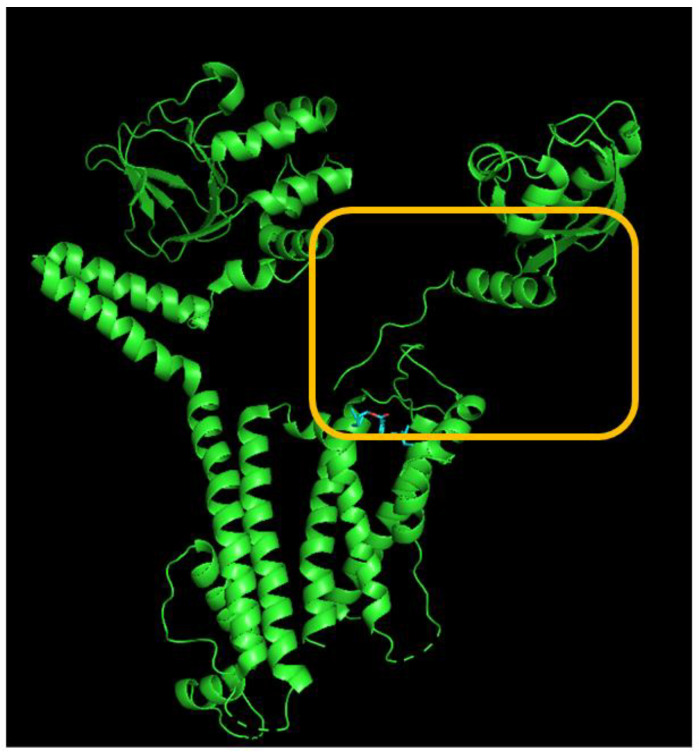

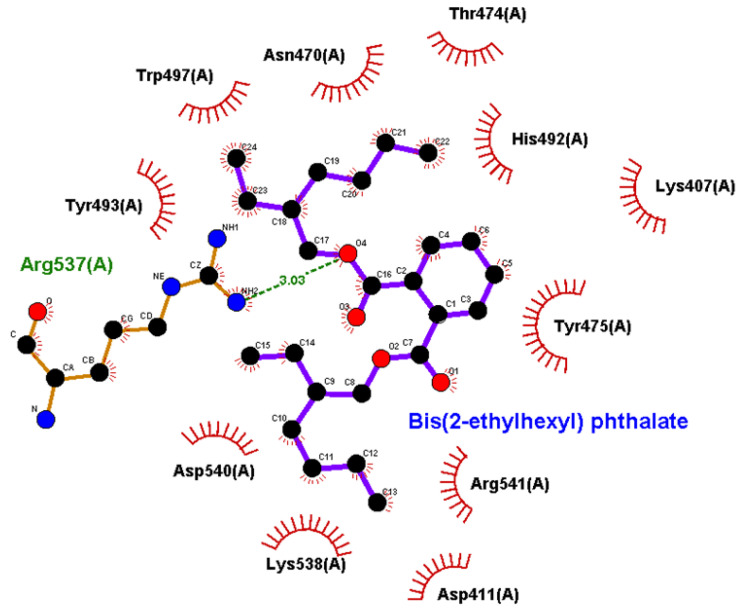
Docking interaction between the di(2-ethylhexyl)-phthalate (DEHP) molecule and the HERG channel. The left graph refers to the docking prediction between DEHP and the HERG channel. The protein structure of HERG was acquired from PDB (PDB ID: 5VA1), while the chemical structure of DEHP was from PubChem (compound CID: 8343). On the upper part, the yellow box indicates a snapshot, illustrating the hydrophobic interactions and hydrogen bond formation between DEHP and the channel shown in the lower part. Note that the red arcs, with spokes directed towards the ligand (such as the DEHP molecule), indicate the presence of hydrophobic contacts. The green dashed line represents the formation of hydrogen bond, with a distance of 3.00 Å.

We carried out a detailed analysis of the atomic interaction between the HERG protein and DEHP using PyRx software. Figure 4 illustrates the predicted docking sites of the DEHP molecule. Notably, during the docking process with the HERG channel, DEHP was noted to establish a hydrogen bond with residue Arg537 with a distance of 3.03 Å. Furthermore, DEHP exhibited hydrophobic interactions with several residues, including Lys407, Asp411, Asn470, Thr474, His492, Tyr493, Trp497, Arg541, Lys538, Asp540, and Arg541. These findings suggest a strong binding affinity between DEHP and the amino acid residues of the HERG channel, estimated at −5.9 kcal/mol. This interaction predominantly occurs in the vicinity of the transmembrane region, specifically around positions 496–516 or 521–541 of the channel. The predicted interaction raises concerns about the potential impact on DEHP-mediated alterations in the magnitude and gating kinetics of *I*_K(erg)_ [8].

Taken together, these results suggest that the inhibition of K_erg_ channels by DHEP or other chemically related compounds may play a role in the changes observed in the functional activities of pituitary cells, including processes like hormonal release, assuming that similar effects can be replicated in a live, in vivo context [8].

*C.*  *M-type K^+^ current (I_K(M)_)*

The *KCNQ2*, *KCNQ3*, and *KCNQ5* genes are responsible for encoding the core subunits of the K_V_7.2, K_V_7.3, and K_V_7.5 channels, respectively [73,74]. These K^+^ channels, when activated, give rise to the *I*_K(M)_, which is found in various electrically excitable cells, including pituitary cells [74,75,76]. This current is characterized by its low threshold voltage activation and displays a slow activation and deactivation profile [74,75]. The regulation of *I*_K(M)_ has garnered considerable interest as an adjunctive therapeutic strategy for addressing neurological disorders marked by excessive neuronal activity. These disorders encompass conditions such as cognitive dysfunction, neuropathic pain, and epilepsy [76,77]. A recent study demonstrated that positron emission tomography can enable the early detection of rapid eye movement sleep behavioral disorder and Parkinson’s disease [78]. It remains to be determined whether this imaging technique can detect dysfunction in the amplitude or gating of *I*_K(M)_. Furthermore, it is believed that the magnitude of *I*_K(M)_ plays a role in regulating the availability of Na_V_ channels during extended periods high-frequency firing [79,80,81].

**1.**  **Solifenacin (SOL, Vesicare^®^, (R)-1-phenyl-3-(1-piperidin-4-ylpropyl)oxy-1,1-diphenyl-4-ylbutan-1-amine)**

SOL, a member of isoquinoline, has been viewed as an oral anticholinergic (i.e., a competitive muscarinic [M_1_ and M_3_] receptor antagonist) and antispasmodic agent used to treat the symptoms of overactive bladder, neurogenic detrusor overactivity, or urinary incontinence [82,83,84]. It has been reported to be a muscarinic (M_2_ and M_3_) receptor antagonist that has anticholinergic effects, such as causing relaxation of the detrusor muscle in urinary bladder [85].

Earlier clinical investigations have revealed the efficacy and safety of the antimuscarinic, SOL, for treating patients with overactive bladder or neurogenic detrusor overactivity [82,84,85]. However, recent evidence has been reported to demonstrate that the treatment with SOL could be linked to an increased risk of impairment in cognitive functions [82,86,87]. It is therefore pertinent to reappraise the mechanism of SOL actions on electrical behaviors in varying excitable cells, given its growing clinical use.

Many types of anterior pituitary cells have been shown to secrete acetylcholine [88]. Earlier studies have also revealed that pituitary GH_3_ cells could exhibit the activity of muscarinic receptors and that muscarinic agonists were able to inhibit hormonal secretion through a reduction in intracellular cyclic AMP [89]. In these pituitary cells, the binding of acetylcholine to the M_2_-muscarinic receptor might induce a weak stimulation on the hydrolysis of phosphatidylinositol 4,5-bisphosphate [90].

A previous report has shown that in pituitary GH_3_ cells, during exposure to SOL, the *I*_K(M)_ amplitude elicited upon membrane depolarization was concentration-dependently increased, with an EC_50_ value of 0.34 μM [9]. The activation time course of *I*_K(M)_ concurrently became shorted and the value of the dissociation constant (K_D_) obtained on the basis of minimal reaction scheme was estimated to be 0.55 μM. The value of EC_50_ required for a SOL-mediated effect on *I*_K(M)_ was similar, reflecting that SOL has the propensity to bind to the open or activated state of the channel. There was a leftward shift in the quasi-steady-state activation curve of *I*_K(M)_ in the presence of SOL. The strength of voltage-dependent hysteresis of *I*_K(M)_ activated by isosceles-triangular ramp pulse became increased during cell exposure to SOL. Furthermore, the K_M_-channel activity was elevated by the addition of SOL, without affecting the single channel conductance of the channel. However, the mean open time of the KM channel after exposure to SOL was increased. Under current-clamp conditions, the firing frequency of spontaneous APs present in GH_3_ cells was found to be effectively decreased in the presence of SOL. Collectively, findings from these results provide an unanticipated and yet non-canonical ionic mechanism through which the SOL molecule can interact with K_M_ channel to enhance whole-cell *I*_K(M)_ and, consequently, diminish the firing rate of spontaneous APs [9]. It appears that whether the effects of SOL (or other structurally similar compounds like darifenacin) on an overactive bladder or neurogenic detrusor overactivity [83,85,91] are related to its enhanced actions on K_M_ channel activity, warrants further investigations, despite its high-affinity binding to muscarinic receptors [85].

**2.**  **Kynurenic acid (KYNA, 4-hydroxyquinoline-2-carboxylic acid)**

KYNA is a naturally occurring product of the normal metabolism of amino acid L-tryptophan that has been reported to inhibit the N-methyl-D-aspartate receptor (NMDAR) and nicotinic α_7_ receptors [92]. This compound, together with L-kynurenine, is thought to be an endogenous metabolite of L-tryptophan known to block NMDAR, and it has been frequently shown to exert neuroprotective or anticonvulsant properties in the brain [92,93,94,95,96]. This compound has been disclosed to inhibit NMDARs at the glycine-binding site, and it can noncompetitively inhibit the α_7_-nicotinic acetylcholine receptor, and through this action, it might modulate glutamate release presynaptically [62,97]. For instance, as administered systemically, a KYNA analog (SZR104) was shown to decrease population spike activity from the pyramidal layer of area CA1 of the hippocampus [93].

Previous studies have shown that the reduction in the astrocytic formation of KYNA could enhance glutamatergic tone in the hippocampus as well as cognitive abilities and synaptic plasticity [94,98,99,100]. KYNA is also recognized to be a target molecule in neuroendocrinology [100]. The KYNA derivatives have been increasing noticed to exert various biological actions [101]. Earlier work has also shown that KYNA-induced hypotension is strongly linked to the stimulation of *I*_K(M)_ magnitude [11]

Interestingly, recent studies have demonstrated that GH_3_-cell exposure to KYNA can result in the stimulation of *I*_K(M)_ with an EC_50_ value of 18.1 μM. The EC_50_ value of KYNA-stimulated *I*_K(M)_ appeared to be lower than that for its inhibition of NR1a/NR2A receptors or AMPA-evoked currents [10]. The relationship of *I*_K(M)_ conductance versus membrane potential during cell exposure to KYNA was noted to produce a leftward shift along the voltage axis by approximately 4 mV [10]. Therefore, it is anticipated to be a pertinent link between KYNA effects on endocrine or neuroendocrine cells and the stimulatory effect on *I*_K(M)_ magnitude.

In addition to the increased *I*_K(M)_ amplitude, the presence of KYNA can shorten the activation time constant of the current. Stimulation of *I*_K(M)_ caused by KYNA is thus not instantaneous but develops over time as K_M_ channels open upon membrane depolarization, thereby leading to an increase in current activation. In keeping with these observations, single-channel current recordings were found to prolong the mean open time of K_M_ channels in the presence of KYNA. Therefore, the increase in both open-state probability and mean open time of the K_M_ channels produced by KYNA or its amide derivatives would be responsible for the increase in macroscopic *I*_K(M)_ carried through these channels, despite their ineffectiveness in changing the single-channel amplitude. In this regard, KYNA or its structurally similar compounds, would be expected to be valuable tools for probing the structure and function of K_M_ channels [102]

*D.*  *Hyperpolarization-activated cation current (I_h_)*

The hyperpolarization-activated cation current (*I*_h_), commonly known as the “funny current” (*I*_f_) and historically referred to as “queer current” in some literature, plays a vital role in regulating repetitive electrical activity in cardiac cells, various types of central neurons, and endocrine or neuroendocrine cells [80,103,104,105,106,107]. This type of ionic current exhibits unique characteristics, including slow voltage-dependent activation kinetics and a mixed Na^+^/K^+^ current that flows inwardly, and it can be blocked by CsCl or ivabradine [104,106,108]. Activation of *I*_h_ may lead to depolarization of the resting potential, reaching the threshold required for generating or triggering an AP. Consequently, it influences pacemaker activity and impulse propagation in electrically active cells [103,107,108,109].

Additionally, the inwardly directed *I*_h_ is activated by sustained hyperpolarization and is known for its gradual activation in a time- and voltage-dependent manner [104]. This can lead to persistent, activity-dependent adjustments in membrane excitability in diverse types of excitable cells [104,105,106,107,110]. Furthermore, the unique voltage-dependent hysteresis of *I*_h_ evoked by double triangular ramp pulse has been demonstrated [108,111,112,113]. The *I*_h_ is mediated by channels encoded by members of the hyperpolarization-activated cyclic nucleotide-gated (*HCN*) gene family, and earlier studies have demonstrated that the activity of these channels underlies the ionic mechanisms associated with both convulsive disorders and inflammatory pain disorders [80,114,115,116,117].

**1.**  **Carisbamate (CRS, RW1-333369, Vimpat^®^, (S)-2-Oxo-1-pyrrolidineacetamide)**

CRS, a bioactive, orally administered neuromodulator, has been shown to be beneficial for the treatment of different types of convulsive disorders, including drug-resistant focal epilepsy and partial-onset seizure [118,119,120]. CRS is thought to work by affecting the activity of certain neurotransmitters in the brain, helping to reduce the occurrence of seizures [13]. This compound was also reported to be effective in the treatment of alcoholism [121]. Another study demonstrated that CRS prevented the development and production of epilepsy-like discharges and exerted a neuroprotective effect after epilepticus-like injury [108].

A recent study has produced an intriguing result, demonstrating that CRS can induce an inhibitory effect on *I*_h_ intrinsically in GH_3_ cells, and the extent of this effect varies with the applied concentration [12]. Using the modified Hill equation, the IC_50_ value required for CRS to suppress the *I*_h_ amplitude as seen in GH_3_ cells was estimated to be 38 μM. With the use of double triangular ramp pulse, the voltage-dependent hysteresis of *I*_h_ can be robustly activated. When the GH_3_ cells were continually exposed to CRS, the hysteretic strength of *I*_h_ evoked by the inverted triangular ramp pulse became progressively decreased [12]. The anticipated docking interaction between CRS and a model of the *HCN* channel also underscores CRS’s capacity to form hydrogen bonds and engage in hydrophobic interactions with amino acid residues in the *HCN* channel [12]. Therefore, in addition to the inhibition of *I*_Na_, CRS can interact directly with the *HCN* channel to alter the magnitude, gating kinetics, and hysteretic strength of *I*_h_ present in pituitary cells. The extent to which CRS-mediated inhibition of *I*_h_ affects brain or endocrine function requires further investigation.

**2.**  **Cannabidiol (CBD, 2-[(1R,6R)-3-methyl-6-prop-1-en-2-ylcyclohex-2-en-1-yl]-5-pentylbenzene-1,3-diol)**

CBD is a non-psychoactive cannabinoid derived from the Cannabis plant, known for its potential therapeutic and medicinal properties. It is among over 100 cannabinoids present in the plant and has been demonstrated to be effective at treating various medical conditions, such as epilepsy, bipolar disorder, inflammation, and cancer [122,123,124]. Recent work has demonstrated that CBD can modify the activity in the hypothalamic–pituitary–adrenal axis [125,126].

A current study showed the effectiveness of CBD in suppressing the magnitude of *I*_h_ in GH_3_ cells and in increasing the activation time constant of the current [14]. The IC_50_ value for CBD-mediated inhibition of *I*_h_ was calculated to be 3.3 μM, and the decrease was reversed by oxaliplatin. In addition to stimulating electroporation-indued currents, oxaliplatin, a platinum-based chemotherapeutic agent, was reported to activate *I*_h_ [127]. The quasi-steady-state activation curve of *I*_h_ was shifted in the leftward direction with no changes in the steepness of the curve in the presence of CBD. This compound also diminished the strength of voltage-dependent hysteresis on *I*_h_ elicited by double triangular ramp pulse [14]. Findings from recent results suggest that CBD’s modification of *I*_h_ does not depend on binding to cannabinoid or opioid receptors. This action may have a significant impact on the functional activities of electrically active cells occurring in vitro or in vivo.

*E.*  *Large-conductance Ca^2+^-activated K^+^ (BK_Ca_) channel*

The large-conductance Ca^2+^-activated K^+^ (BK_Ca_ or BK) channels (KCa1.1, *KCNMA1*, *Slo1*) belong to the voltage-gated K^+^ channel family. They are activated by an increase in the intracellular Ca^2+^ concentration, membrane depolarization, or a combination of both [128,129]. Activation of BK_Ca_ channels can lead to the flow of large amounts of K^+^ ions across the cell membrane. With its high-conductance state and a single-channel conductance of approximately 150–250 pS, the BK_Ca_ channel is also considered a maxi- or large-K^+^ channel. This family of K^+^ channels is functionally expressed in pituitary cells, and its activity can affect the magnitude of whole-cell Ca^2+^-activated K^+^ currents (*I*_K(Ca)_) in pituitary cells, consequently impacting the membrane potential and stimulus–secretion coupling of these cells. How the activity of BK_Ca_ channels present at the level of the pituitary gland could be implicated in the intervertebral disk degeneration [130] remains to be clarified.

**1.**  **Rufinamide (RFM, Banzel^®^, Inovelon^®^, ethyl 1-(2,6-difluorophenyl)-1H-1,2,3-triazole-4-carboxylate)**

RFM is recognized as a unique anticonvulsant drug, because, as a triazole derivative, its structure is dissimilar to other currently marked antiepileptic drugs [131,132]. It is increasingly being used in combination with other medications and therapies to treat Lennox–Gaustaut syndrome, severe epileptic encephalopathy, and other seizure disorders [133,134,135,136,137]. Lennox–Gaustaut syndrome is a rare and severe form of epilepsy that typically begins in childhood, and it is characterized by multiple types of seizures and intellectual and developmental disabilities.

**Figure 5:**
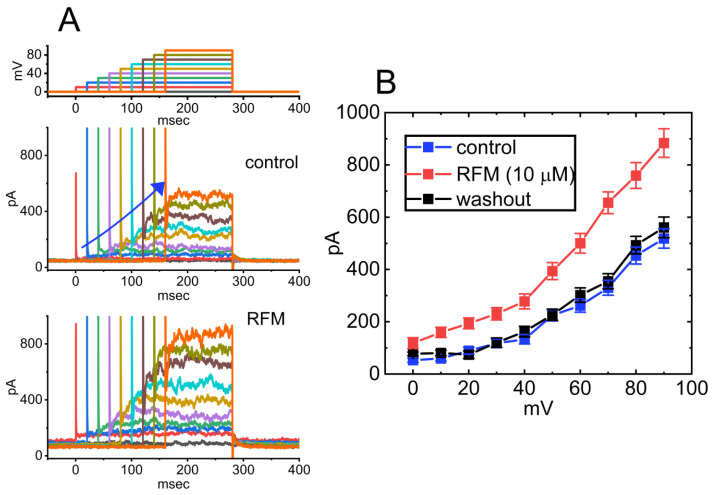
Effect of rufinamide (RPM) on mean current–voltage (*I-V*) relationships of Ca^2+^-activated K^+^ current (*I*_K(Ca)_) identified in GH_3_ cells. (**A**) Representative current traces obtained in the absence (upper) and presence (lower) of 10 μM RFM. The uppermost part shows the voltage-clamp protocol applied. The potential traces labeled in different colors correspond to current ones acquired without or with RPM presence. The duration of each depolarizing step is different for better illustrations, and the blue solid arrow indicates the outwardly rectifying properties of *I*_K(Ca)_ with increasing positive voltage. (**B**) Mean *I-V* relationships of *I*_K(Ca)_ amplitude acquired in the control (blue squares), during exposure to 10 μM RPM (red squares), and following washout of RFM (black squares) (mean ± SEM for each point). Current amplitudes were measured at the end of each depolarizing step. This figure is adapted from Lai el al. [15] and published under the terms and conditions of the Creative Commons Attribution (CC BY) license.

Although the mechanism of RFM action as an antiepileptic drug is still unclear, RFM was reported to modulate the activity of Na_V_ channels by prolonging the inactive state of these channels [138,139]. Recent studies have also shown that RFM can interact with BK_Ca_ channels to enhance whole-cell Ca^2+^-activated K^+^ currents (*I*_K(Ca)_) effectively. As shown in Figure 5, the application of 10 μM RPM significantly increased the amplitude of *I*_K(Ca)_ across the entire voltage-clamp step. The effective EC_50_ value of RFM required for stimulating *I*_K(Ca)_ was estimated to be 3.9 μM, with a Hill coefficient of 1.2 [15]. The maximum plasma concentrations of RFM at dosages of 10 mg/kg/day and 30 mg/kg/day have been reported as 4.01 μg/mL (16.8 μM) and 8.68 μg/mL (36.4 μM), respectively [140]. Consequently, the EC_50_ value is observed to be within the range of clinically achieved concentrations.

Additionally, the docking study showed that RFM can bind to the intracellular domain of the K_Ca_1.1 channel at certain amino acid residues and that the RFM-induced docking site is not located in the pore regions of the channels [15]. Indeed, in pituitary GH_3_ cells, the addition of RFM to the cytosolic surface of the detached patch of membrane resulted in the enhanced activity of BK_Ca_ channels, with no modification in single-channel conductance of the channel [15]. The mean closed time of BK_Ca_ channels was decreased by the application of RFM to the cytosolic leaflet of the channel. Overall, aside from its ability to block *I*_Na_, similar to riluzole (as demonstrated previously) [129], RFM has been shown to effectively enhance the activity of BK_Ca_ channels within excitable cells in in vivo settings.

**2.**  **QO-40 ((5-(chloromethyl)-3-(naphthalen-1-yl)-2-(trifluoromethyl)pyrazolo [1,5-a]pyrimidin-7(4 H)-one)**

QO-40 is a highly pure, synthetic, and biologically active compound. This compound has been previously reported to enhance KCNQ2/KCNQ3 heteromeric currents expressed in *Xenopus* oocytes [132]. QO58-lysine, a compound structurally similar to QO-40, can also activate neuronal KCNQ channels and exert antinociceptive effects on inflammatory pain [31]. The QO-58-induced amelioration of inflammatory pain observed in rodents was previously viewed as being accompanied by the activation of KCNQ-encoded K^+^ currents [31,141].

In a recent study [16], as pituitary GH_3_ cells were exposed to QO-40, the magnitude of *I*_K(Ca)_ was observed to be notably increased, with an EC_50_ value of 2.3 μM. QO-40-stimulated *I*_K(Ca)_ was attenuated by further addition of paxilline, yet not by linopirdine or TRAM-34. It is worth noting that paxilline is a tremorgenic mycotoxin known to suppress the activity of BK_Ca_ channels [128], while linopirdine inhibits the *I*_K(M)_ magnitude, and TRAM-34 can suppress the activity of intermediate-conductance Ca^2+^-activated K^+^ channels [142]. In inside-out single-channel recordings, it was observed that QO-40 not only produced a 14 mV shift towards a less positive potential in the steady-state activation curve of BK_Ca_ channels but also increased the gating charge by 1.4-fold. However, it is important to highlight that QO-40 did not alter the single-channel conductance of the channel, despite causing a reduction in the mean closed time of BK_Ca_ channels when it was present. Additionally, with the long-lasting isosceles-triangular ramp pulse, cell exposure to QO-40 enhanced the voltage-dependent hysteretic strength of BK_Ca_ channels. Although the detailed mechanism of the stimulatory actions of QO-40 on BK_Ca_ channels is not yet known, experimental observations suggest that QO-40 can enhance the activity of BK_Ca_ channels in a voltage-dependent manner [16]. As a result, its interaction with the BK_Ca_ channel can vary significantly based on several factors such as the resting potential, AP firing pattern, the concentration of QO-40 used, or any combination of these variables.

The maximal concentration of QO-58, a synthesized compound that is structurally similar to QO-40, following oral administration at 25, 50, or 100 mg/kg, has been reported to reach 8.25, 16.29, or 18.27 mg/liter (approximately 18.6, 37, or 41 μM), respectively [143]. In this scenario, the stimulatory effect of QO-40 on BK_Ca_ channels would be of pharmacological or therapeutic relevance, as this compound at lower concentrations is effective at stimulating *I*_K(Ca)_ and enhancing BK_Ca_ channel activity. However, it remains to be answered whether the rank order for QO-40 or other chemically related agents in activating BK_Ca_ channels would share a similar magnitude for their stimulation of neuronal *KCNQ* currents.

## 3. Conclusions

This paper does not focus voltage-independent currents, such as those mediated by transient receptor potential (TRP) channels, including TRPC, TRPM, and TRPV [144]. Instead, it provides a proof-of-concept for understanding the pathophysiological and pharmacological roles of pituitary cells, with a particular emphasis on the functionality of voltage-gated ion channels. Additionally, this paper explores how specific drugs or compounds affect these intrinsic ionic currents within pituitary cells. These insights are crucial for regulating the function of electrically active cells and advancing our understanding of pituitary neuroendocrine tumors (PitNets). Notably, rats can also develop PitNets, or pituitary tumors, in their pituitary glands, much like humans and some other animals.

It is important to note that the current pituitary cell lines primarily used in research are derived from rats, such as GH_3_ cells, GH_4_C_1_ cells, MMQ, R1220, and AtT-20 cells [3,5,10,45,145,146]. Detailed descriptions of these cell lines can be found in Table 2. However, there are limited reports on immortalized human pituitary cell lines. Whether the electrical properties observed in these cell lines, as well as their responses to various drugs, are preserved in human pituitary cells remains an area that requires further in-depth investigation. In addition to pituitary cells, other endocrine cell types―such as those responsible for insulin and glucagon secretion, as well as Leydig cells―have also been shown to exhibit similar ionic currents [1,4,146,147,148,149,150]. However, further research is needed to determine whether the drugs or compounds mentioned here impact the electrical activity of these different endocrine cell types and their functional implications. Understanding the regulation of the ion currents discussed in this paper is crucial for unraveling the molecular mechanisms underlying PiNets and their potential for advancing therapeutic approaches [145,151,152,153,154].

In clinical practice, it is common to observe that many patients with PitNets develop tumors in other endocrine glands, leading to the occurrence of an endocrine tumor syndrome known as multiple endocrine neoplasia type 1 (Wermer syndrome) [89,118,126,154,155,156,157,158,159,160]. Computerized tomography-guided radiofrequency ablation has become a key treatment option for PiNets’ removal, helping to minimize the risk of damage to surrounding deep brain tissues [126,151]. Furthermore, the development of medications designed to prevent the recurrence of such tumors, as well as other forms of multiple endocrine neoplasia and neuroendocrine tumors like small cell lung carcinoma, will be a crucial area for future research [18,145,154,155]. At the same time, it is also an important task to conduct in-depth investigations of potential genetic abnormalities in patient samples of these PiNets [161]. Therefore, the comprehensive research presented in this paper will play a crucial role in advancing our comprehension of the origin and management of these conditions.

Although techniques like polymerase chain reaction (PCR) and Western blotting can be used to measure gene and protein expression abnormalities, respectively, in PitNets, performing patch-clamp experiments on different pituitary cells enables direct investigation of the biophysical properties of individual or whole ionic currents across the cell membrane, as well as changes in membrane potential, as presented herein. Another advanced and innovative technique, such as the automatic patch-clamp technique provided by Sophion Bioscience (http://sophion.com, accessed on 14 April 2025), is also an alternative option for conducting cell electrophysiology studies on pituitary cells. Nonetheless, it remains a crucial approach for functional studies. Additionally, performing direct measurements on PitNets’ organoid [152] will open up further opportunities for detailed pituitary research in the future.

## Figures and Tables

**Table 1:** A brief description of the transmembrane ionic currents known to exist in pituitary cells, along with the effects of various drugs or compounds with their respective chemical structures on these ion currents demonstrated in this paper.

Ionic Current	Chemical or Drug	Abbreviated Name	Chemical Structure *	References **
*I*_Na_	GV-58	NA	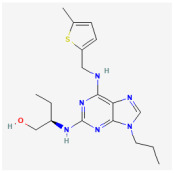	[4]
	Esaxerenone	ESAX	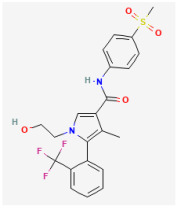	[5]
*I*_K(erg)_	Risperidone	NA	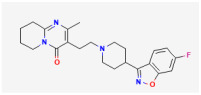	[6,7]
	Di(2-ethylhexyl)-phthalate	DEHP	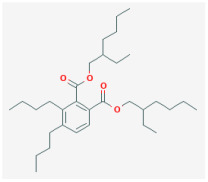	[8]
*I*_K(M)_	Solifenacin	SOL	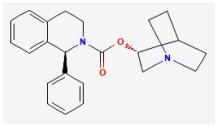	[9]
	Kynurenic acid	KYNA	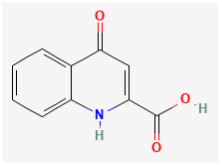	[10,11]
*I*_h_	Carisbamate	CRS	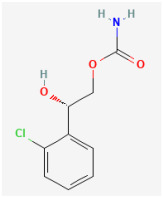	[12,13]
	Cannabidiol	CBD	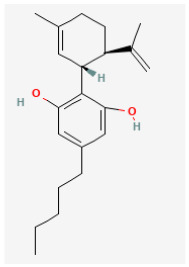	[14]
BK_Ca_ channel	Rufinamide	RFM	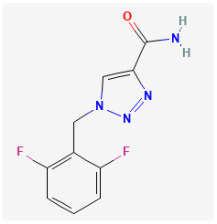	[15]
	QO-40	NA ***	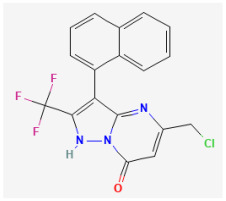	[16]

* Two-dimensional chemical structure acquired at https://pubchem.ncbi.nlm.nih.gov/ which was accessed on 10 April 2025. ** The numbers in the square brackets represent the reference number. *** NA = non-available.

**Table 2:** Pituitary cell lines cited in this article, along with their sources and links to websites for more detailed information.

Cell Line	ATCC * Website	BCRC * Website	ScienCell^TM^ Website
AtT-20	https://www.atcc.org/products/ccl-89 (CCL-89)	https://catalog.bcrc.firdi.org.tw/BcrcContent?bid=60244&rowid=1	
GH_3_	https://www.atcc.org/products/ccl-82.1 (CCL-82.1)	https://catalog.bcrc.firdi.org.tw/BcrcContent?bid=60015&rowid=1	
GH_4_C_1_	https://www.atcc.org/products/ccl-82.2 (CCL-82.2)	NA	
MMQ	https://www.atcc.org/products/crl-10609 (CRL-10609)	NA	
R1220			https://sciencellonline.com/rat-pituitary-cel

* ATCC stands for the American type culture collection (Manassas, VA), while BCRC refers to the Bioresource Collection and Research Center (Hsinchu, Taiwan). NA = non-available (accessed on 10 March 2025).

## Data Availability

Data are available by request.

## Abbreviations

The following abbreviations are used in this manuscript:

AP	action potential
BK_Ca_ channel	large-conductance Ca^2+^-activated K^+^ channel
HERG channel	human *erg* K^+^ channel
*I*_h_	hyperpolarization-activated cationic current
*I*_K(Ca)_	Ca^2+^-activated K^+^ current
*I*_K(erg)_	*erg*-mediated K^+^ current
*I*_K(M)_	M-type K^+^ current
*I*_Na_	voltage-gated Na^+^ current
*I*_Na(P)_	persistent Na^+^ current
K_erg_ channel	*erg*-mediated K_V_ channel
Na_V_ channel	voltage-gated Na^+^ channel
PitNet	pituitary neuroendocrine tumor
